# Effect of Neurexan on the pattern of EEG frequencies in rats

**DOI:** 10.1186/1472-6882-12-126

**Published:** 2012-08-16

**Authors:** Wilfried Dimpfel, Kerstin Roeska, Bernd Seilheimer

**Affiliations:** 1 Justus-Liebig-University Giessen, NeuroCode AG, Sportparkstrasse 9, Wetzlar, 35578, Germany; 2 Biologische Heilmittel Heel GmbH, Dr.-Reckeweg-Str. 2-4, Baden Baden, 76532, Germany

**Keywords:** Central nervous system, Stress-related disorders, Multitargeted, Multicomponent, Brain waves, Electroencephalography

## Abstract

**Background:**

Various medications of natural origin have effectively treated stress-related disorders, such as sleep disturbances and agitated conditions. The efficacy of Neurexan, a multicomponent, low-dose medication, has been demonstrated in observational studies, but its exact mechanism of action has not been determined.

**Methods:**

To characterize the effects of Neurexan on the central nervous system, we analyzed the spectral frequencies of field potentials in four rat brain areas after a single oral administration of Neurexan. Different doses of Neurexan were tested within a crossover design, and effects were compared with vehicle control.

**Results:**

Significant effects were observed with 0.5 tablets of Neurexan, predominantly on δ- and θ-waves in the frontal cortex and reticular formation (*P* < 0.01). In the reticular formation, significant changes of δ- and θ-waves occurred as early as during the first hour after administration. The time course revealed a significant and longer-lasting increase of δ- and θ-waves in the frontal cortex and reticular formation, whereas other spectral frequencies were only transiently affected in the frontal cortex, reticular formation, and striatum.

**Conclusion:**

In conclusion, this study demonstrated that the low-dose medication Neurexan influences central nervous system activity in rats. The resulting electroencephalographic profile of Neurexan shows several similarities with those of other calming agents, such as Valeriana and Passiflora, suggesting a potential benefit of Neurexan for patients with stress-related disorders. Moreover, this report demonstrates that electroencephalographic signatures are also valid biomarkers for the assessment of low-dose medications, such as Neurexan.

## Background

Stress-related disorders, such as mild anxiety, nervousness, and insomnia, are widespread among the population. For example, the prevalence of anxiety disorders and insomnia reaches 16.6% and 26%, respectively [1,2]. Currently used medications include benzodiazepines and serotonin-specific reuptake inhibitors. However, these medications can be accompanied by considerable adverse effects, such as cognitive impairment and dependency [3-5]. For the treatment of stress-related disorders, self-medication is often practiced, and there is a strong association between stress and alcohol consumption [6], leading to the known potential consequences for physical and psychological health.

Clinical studies have documented that complementary and alternative medicine effectively treats stress-related disorders, such as sleep disturbance and anxiety-related conditions, without serious adverse effects [7,8]. For example, Neurexan is a multicomponent drug constituted of highly diluted natural, mainly plant, extracts. It is recommended to treat mild sleep disturbances and agitated conditions [9].

The effectiveness of the single components of Neurexan (*passiflora incarnata* [Passiflora], *avena sativacoffea arabica*, and *zincum isovalerianicum*) has been demonstrated in human and animal studies. Three randomized controlled trials demonstrated the anxiolytic efficacy of Passiflora as monotherapy [10,11] and as part of an herbal combination [12]. Animal studies demonstrated the sedative effects of *avena sativa*[13] and increased sleep intensity by diluted preparations of *coffea cruda*[14]. In a retrospective analysis, a potential beneficial effect of *zincum isovalerianicum* in patients with restless legs syndrome could be observed [15].

Although these components are less concentrated in Neurexan than in the reference drugs tested in the previously mentioned studies, Neurexan is effective for the treatment of moderate insomnia and nervousness. In two observational studies, Neurexan was demonstrated as a well-tolerated alternative to conventional *valeriana officinalis* (Valeriana)–based therapies for the treatment of moderate insomnia and nervousness [16,17].

As a multicomponent medication, Neurexan presumably acts on different targets, and the resulting net effect on the central nervous system has not been characterized. During the past decade, electroencephalography (EEG) has provided surrogate measures of drug efficacy of psychoactive drugs. The EEG signature obtained from the spectra of oscillation frequencies may differ between baseline and experimental conditions. Therefore, an EEG signature might be seen as a biomarker [18].

Biomarkers are increasingly used to both predict the clinical response to treatment and characterize the mechanism of action [19]. For instance, changes of EEG signatures observed after administration of experimental compounds can be assigned to the interference of drugs with certain neurotransmitter systems, such as the cholinergic, dopaminergic, and noradrenergic systems [20-23]. Moreover, different drug categories, such as antidepressive, anticonvulsive, analgesic, neuroleptic, stimulatory, narcotic, sedative, and hallucinogenic drugs, result in a reproducible and disease-specific EEG signature. Therefore, unknown compounds could be classified into distinct drug categories by discriminant analysis of EEG data [24]. More important, the conservation of brain structure and neurobiological features across mammalian species allows the translatability of results [18].

In this report, we asked if the multicomponent, low-dose medication Neurexan induces an EEG signature that explains its calming effects. To answer this question, we characterized the effect of different doses of Neurexan on the EEG of four rat brain areas during 5-hour experiments.

## Methods

### Drugs

Neurexan (Heel GmbH, Baden-Baden, Germany) is an over-the-counter medication constituted of highly diluted ingredients. One tablet consists of 0.6 mg *passiflora incarnata* D2, 0.6 mg *avena sativa* D2, 0.6 mg *coffea arabica* D12, 0.6 mg z*incum isovalerianicum* D4, 1.5 mg magnesium stearate, and 300 mg lactose-monohydrate (D indicates dilution). All components are prepared according to the *European Pharmacopoeia*[25].

Neurexan is recommended for the treatment of mild sleep disturbances and agitated conditions [9]. In animal experiments, Neurexan was dissolved in 0.9% NaCl (1 mL/kg body weight). The applied doses of Neurexan (0.25, 0.50, or 1 tablet per animal) correspond to the 6- to 12-fold human equivalent dosage [26]. Neurexan or vehicle controls (1 mL/kg 0.9% NaCl) were orally administered as a single dose. Other drugs used as comparators in discriminant analyses were described earlier [24].

### Animals

Eight 8-month-old Fisher 344 rats (Charles River Laboratories, Sulzfeld, Germany) were kept at the preclinical study center (NeuroCode AG, Wetzlar, Germany) in sterile filter-top cages on a reversed day-night cycle and provided with food and water ad libitum. Animal procedures were performed according to German health guidelines and animal protection laws and were approved by the Regional Commission Gießen (GI 21/4-Nr.66-2009, Regierungspräsidium Gießen, Schanzenfeldstraße 8, 35578 Wetzlar). For surgery, animals were anesthetized with an intramuscular injection of 10% ketamine hydrochloride (1 mL/kg). Four concentric bipolar steel electrodes were then implanted in the left hemisphere (3 mm lateral) by stereotactic surgery. The anterior coordinates were 12.2, 5.7, 9.7, and 3.7 mm for the frontal cortex, hippocampus, striatum, and reticular formation, respectively [27]. A plate carrying four bipolar SNF100 neurological electrodes (Rhodes Medical Instruments Inc, Summerland, CA), a 5-pin plug, and a transmitter (26 × 12 × 6 mm; 5.2 g) was fixed on the skull by three steel screws and dental cement.

### EEG recording

For the recording of EEG signals, cages were placed in a copper-shielded room. As described earlier, signals were transmitted by a wireless radio-telemetric system (Rhema Labortechnik, Hofheim, Germany) and processed to give power spectra of 0.25-Hz resolution [28]. After automatic artifact rejection, signals were collected in 4-second units and fast Fourier transformed using a Hanning window. Sampling frequency was 512 Hz. Four values were averaged to give a final sampling frequency of 128 Hz, well above the Nyquist frequency. The resulting electrical power spectra were divided into δ-waves (0.8-4.5 Hz), θ-waves (4.75-6.75 Hz), α1-waves (7.00-9.50 Hz), α2-waves (9.75-12.50 Hz), β1-waves (12.75-18.50 Hz), and β2-waves (18.75-35.00 Hz). Spectra were averaged every 3 minutes. For further analysis, periods of 60 minutes were averaged. To exclude circadian effects, all measurements were performed at the same time. During recording, rats were moving freely but could not eat to avoid artifacts.

### Study design

The study began 2 weeks after surgery. Animals were assigned to receive different doses of Neurexan or vehicle control in a crossover design. All eight animals were exposed to one dose per experiment and then received a different dose after a 1-week washout period. In each experiment, a single dose of Neurexan or vehicle control was administered orally after a predrug baseline recording for 45 minutes. Then, after a 5-minute interval for recovery, EEG signals were recorded for 5 hours.

### Statistics

The EEG signals (in μV^2^) were documented in hourly intervals as percentage of baseline signals. Each of the six frequency bands within each brain region was measured, and the data of eight animals were summarized as mean ± SEM. The differences of frequency band changes between Neurexan and vehicle-treated rats were tested for significance using the Wilcoxon Mann–Whitney *U* test. *P* < 0.01 was considered significant.

## Results

**Figure 1:**
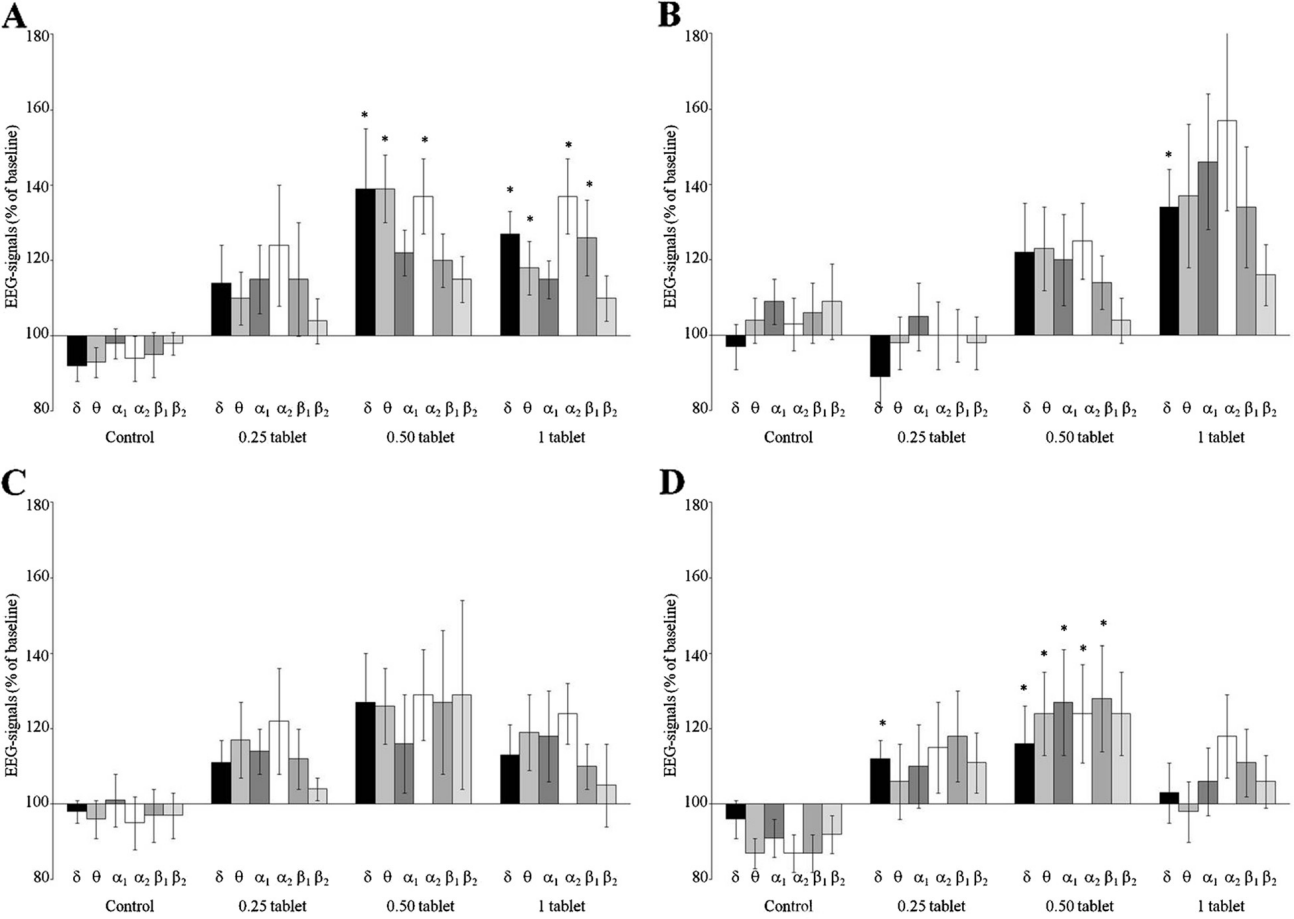
**Effects of control and 0.25, 0.50, and 1 tablet of Neurexan on the spectral frequencies in four rat brain areas (frontal cortex [A], hippocampus [B], striatum [C], and reticular formation [D]).** The encephalographic (EEG) signatures were obtained between 185 and 245 minutes after oral administration of a single dose of Neurexan. Changes of δ-, θ-, α1-, α2-, β1-, and β2-waves are presented as percentage of predrug baseline. The data of eight rats are summarized as mean ± SEM. Significant differences to vehicle controls (0.9% NaCl) are indicated by asterisks (*P* < 0.01).

The dose–response relationship between Neurexan and EEG signatures revealed a predominant effect on δ-, θ-, and α2-waves in the frontal cortex and reticular formation (Figure 1). Changes of spectral frequencies were analyzed between 185 and 245 minutes after oral administration of a single dose of Neurexan. Although changes of spectral frequencies were observed at each dosage (0.25, 0.5, or 1 tablet), most significant changes were found with 0.5 tablets of Neurexan. At this concentration, δ-, θ-, and α2-waves were significantly changed in the frontal cortex when compared with vehicle control (Figure 1A). Moreover, δ-, θ-, and α2-waves and, in addition, α1- and β1-waves were significantly altered in the reticular formation (Figure 1D). In the hippocampus, only δ-waves were significantly affected by treatment with 1 tablet of Neurexan (Figure 1B). Between 185 and 245 minutes, no significant change of spectral frequencies could be observed in the striatum (Figure 1C).

**Figure 2:**
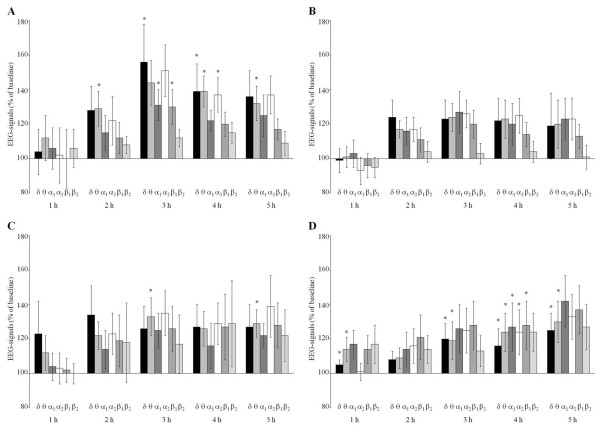
**Time course of the effects of 0.5 tablets of Neurexan on the spectral frequencies in four rat brain areas (frontal cortex [A], hippocampus [B], striatum [C], and reticular formation [D]).** The electroencephalographic (EEG) signatures were obtained during five experimental intervals of 1 hour (1, 2, 3, 4, and 5 hours). Changes of δ-, θ-, α1-, α2-, β1-, and β2-waves are presented as percentage of predrug baseline. The data of eight rats are summarized as mean ± SEM. Significant differences to vehicle controls (0.9% NaCl) are indicated by asterisks (*P* < 0.01).

Over time, 0.5 tablets of Neurexan significantly changed most spectral frequencies in the frontal cortex, reticular formation, and striatum when compared with vehicle control. Effects were predominantly observed on δ- and θ-waves (Figure 2). In the frontal cortex, δ- and θ-waves persistently and significantly increased during the third to fourth hour and during the fourth to fifth hour after administration, respectively (Figure 2A). In the reticular formation, δ- and θ-waves significantly increased during the third to fifth hour and, in addition, during the first hour after administration (Figure 2D). Other spectral frequencies were only transiently affected by Neurexan. In the frontal cortex and reticular formation, α1-, α2-, and β1-waves were significantly increased at single experimental intervals. In the striatum, changes of θ-waves reached significance at two experimental intervals (third and fifth hours, Figure 2C), whereas no significant changes were observed in the hippocampus at this dosage (Figure 2B).

Taken together, Figures 1 and 2 demonstrate that even a single dose of Neurexan significantly modulated the EEG signature of several brain areas when compared with vehicle control. The EEG changes predominantly affected δ-, θ-, and, to a minor extent, α2-waves in the frontal cortex and reticular formation.

## Discussion

Herein, we demonstrate that the low-dose medication Neurexan induces significant changes of rat EEG signatures. Neurexan-induced changes predominantly affect δ- and θ-waves in the frontal cortex and reticular formation, supporting the fact that Neurexan generally exhibits calming effects.

The rat EEG signature was significantly altered after a single oral administration of Neurexan when compared with vehicle control, thus characterizing Neurexan as psychoactive. Changes were observed in the reticular formation as early as during the first hour after administration. This immediate effect differentiates Neurexan from other plant-derived drugs against stress-related disorders that are effective as early as 3 hours after administration [29]. At later time points, EEG signatures of the frontal cortex and striatum, but not the hippocampus, were also affected by Neurexan.

After having established the principal evidence of dose- and time-dependent effects of Neurexan on the central nervous system, we found a predominant increase of δ- and θ-waves, supporting the calming effect of Neurexan in general. Earlier publications have demonstrated that pharmacological interference with neurotransmitter systems results in changes of distinct brain waves. Accordingly, changes of δ-waves correspond to interference with the cholinergic system [20]. The fact that enhancing cholinergic transmission in the reticular formation increases rapid eye movement sleep in several species [30-32] might, therefore, contribute to an explanation of the beneficial effects of Neurexan on sleep disturbance [16]. According to previous studies [21,33], Neurexan-induced changes of α2-waves can be interpreted as inactivation of the dopaminergic system, whereas increases of θ-waves could be related to interference with the norepinephrine α2 receptor, leading to sedation [22].

The effects on EEG signatures in rats have been demonstrated for several different synthetic and plant-derived psychoactive substances, and, more important, the same method of EEG recording was used in these studies [20,24,28]. We were, therefore, able to descriptively compare the net effects of Neurexan on six frequency bands in four rat brain areas with those of other substances. It might not be scientifically adequate to draw direct conclusions from these comparisons, but several similarities between Neurexan and Valeriana or Passiflora could be found (data not shown).

Both Valeriana and Passiflora are present in Neurexan, although at a far smaller concentration. Several clinical trials have demonstrated that Valeriana effectively treats insomnia mostly by reducing sleep latency and improving sleep quality. In two double-blind and placebo-controlled studies, single doses between 400 and 900 mg significantly improved sleep latency and sleep quality when compared with placebo [34,35]. In two randomized, double-blind studies, the effects of Valeriana (600 mg/d) on insomnia were comparable to those of Oxazepam [36,37]. Interestingly, a high dosage of Valeriana (1500 mg Valeriana in combination with 360 mg hops) particularly increased δ-waves in human subjects [38]. However, the concentration of Valeriana in Neurexan is far lower (0.6 mg valerianate of zinc D4 per tablet), but Neurexan has been as effective as Valeriana-based therapies for the treatment of mild to moderate insomnia [16]. Either highly diluted valerianate of zinc might be as effective as high-dose Valeriana or, more probably, the combination of valerian ate of zinc with other components of Neurexan synergistically mediates calming effects.

The second comparator drug generating a similar EEG signature such as Neurexan, Passiflora, exerts anxiolytic effects in mice [39,40] and humans [10-12]. In a double-blind, placebo-controlled trial, Passiflora has been as effective as benzodiazepine in eliminating anxiety symptoms [10]. Doses of 10 mg/kg body weight were effective in mice [39]. In our experiments, the reference drug Passiflora was applied at a dose of 100 mg/kg and predominantly increased θ-activity in the frontal cortex and striatum (data not shown). Likewise, 1200 mg of Passiflora extract strongly increased θ-waves and slightly increased δ-waves in patients with sleep disorders [41]. Concerning this influence on EEG activity, Neurexan containing low-dose Passiflora seems to be comparably effective as the high-dose comparator drug.

We recognize two major limitations of our study. First, the number of animals in our study was relatively small, but the sample size was sufficient to detect significant effects (*P* < 0.01) on the EEG signature in rats. Second, our results are rather descriptive and need to be correlated with the effects of Neurexan on human subjects, even if the translatability of results across mammalian species might be widely accepted [18]. However, one objective of our study was to demonstrate an effect of this low-dose and multicomponent medication on the central nervous system, providing a starting point for further research. Correlation with the effectiveness of Neurexan on moderate insomnia and nervousness has already been provided by two observational studies [16,17]. Moreover, a double-blind, placebo-controlled trial is conducted to evaluate the psychophysiological effects of Neurexan on human subjects in more detail.

## Conclusion

In summary, the current study demonstrated that the multicomponent, low-dose medication, Neurexan, influences neuronal activity in particular brain areas, thereby suggesting that the drug has calming effects. The similarity of Neurexan with other medications with known calming effects suggests that the combination of the lower concentrated components of Neurexan exerts an additive and synergistic effect on neuroactivity. It can be speculated that Neurexan has beneficial effects for patients with stress-related disorders. Moreover, this report demonstrates that EEG signatures are valid biomarkers for the psychoactivity of multicomponent, low-dose medications, such as Neurexan. The outcome gives guidance for further scientific investigations of that drug, to identify the working mechanism and its efficacy in more detail.

## Abbreviations

EEG: Electroencephalography; CNS: Central nervous system; NaCl: Natrium chloride; D: Dilution; SEM: Standard error of the mean; REM: Rapid eye movement.

## Competing interests

Research by W. Dimpfel (NeuroCode AG) was performed on behalf of Biologische Heilmittel Heel GmbH that also provided financial support for editorial assistance and scientific writing services. K. Roeska and B. Seilheimer are employees of Biologische Heilmittel Heel GmbH.

## Authors’ contributions

WD was responsible for the performance of the experiments. KR made substantial contributions to the interpretation of the data and was involved in drafting the manuscript. BS was involved in writing the manuscript and approved the final version. All authors read and approved the final manuscript.

## Pre-publication history

The pre-publication history for this paper can be accessed here:

http://www.biomedcentral.com/1472-6882/12/126/prepub
